# Activated microglia/macrophage whey acidic protein (AMWAP) inhibits NFκB signaling and induces a neuroprotective phenotype in microglia

**DOI:** 10.1186/s12974-015-0296-6

**Published:** 2015-04-19

**Authors:** Alexander Aslanidis, Marcus Karlstetter, Rebecca Scholz, Sascha Fauser, Harald Neumann, Cora Fried, Markus Pietsch, Thomas Langmann

**Affiliations:** Laboratory for Experimental Immunology of the Eye, Department of Ophthalmology, University of Cologne, Kerpener Strasse 62, D-50931 Cologne, Germany; Institute of Reconstructive Neurobiology, University of Bonn, Sigmund-Freud-Straße 25, D-53127 Bonn, Germany; Department of Pharmacology, University of Cologne, Gleueler Straße 24, D-50931 Cologne, Germany

**Keywords:** Activated microglia/macrophage whey acidic protein (AMWAP), Microglia, NFκB, Photoreceptors, Neurodegeneration

## Abstract

**Background:**

Microglia reactivity is a hallmark of neurodegenerative diseases. We have previously identified activated microglia/macrophage whey acidic protein (AMWAP) as a counter-regulator of pro-inflammatory response. Here, we studied its mechanisms of action with a focus on toll-like receptor (TLR) and nuclear factor κB (NFκB) signaling.

**Methods:**

Recombinant AMWAP was produced in *Escherichia coli* and HEK293 EBNA cells and purified by affinity chromatography. AMWAP uptake was identified by fluorescent labeling, and pro-inflammatory microglia markers were measured by qRT-PCR after stimulation with TLR ligands. NFκB pathway proteins were assessed by immunocytochemistry, Western blot, and immunoprecipitation. A 20S proteasome activity assay was used to investigate the anti-peptidase activity of AMWAP. Microglial neurotoxicity was estimated by nitrite measurement and quantification of caspase 3/7 levels in 661W photoreceptors cultured in the presence of microglia-conditioned medium. Microglial proliferation was investigated using flow cytometry, and their phagocytosis was monitored by the uptake of 661W photoreceptor debris.

**Results:**

AMWAP was secreted from lipopolysaccharide (LPS)-activated microglia and recombinant AMWAP reduced gene transcription of IL6, iNOS, CCL2, CASP11, and TNFα in BV-2 microglia treated with LPS as TLR4 ligand. This effect was replicated with murine embryonic stem cell-derived microglia (ESdM) and primary brain microglia. AMWAP also diminished pro-inflammatory markers in microglia activated with the TLR2 ligand zymosan but had no effects on IL6, iNOS, and CCL2 transcription in cells treated with CpG oligodeoxynucleotides as TLR9 ligand. Microglial uptake of AMWAP effectively inhibited TLR4-dependent NFκB activation by preventing IRAK-1 and IκBα proteolysis. No inhibition of IκBα phosphorylation or ubiquitination and no influence on overall 20S proteasome activity were observed. Functionally, both microglial nitric oxide (NO) secretion and 661W photoreceptor apoptosis were significantly reduced after AMWAP treatment. AMWAP promoted the filopodia formation of microglia and increased the phagocytic uptake of apoptotic 661W photoreceptor cells.

**Conclusions:**

AMWAP is secreted from reactive microglia and acts in a paracrine fashion to counter-balance TLR2/TLR4-induced reactivity through NFκB inhibition. AMWAP also induces a neuroprotective microglial phenotype with reduced neurotoxicity and increased phagocytosis. We therefore hypothesize that anti-inflammatory whey acidic proteins could have a therapeutic potential in neurodegenerative diseases of the brain and the retina.

**Electronic supplementary material:**

The online version of this article (doi:10.1186/s12974-015-0296-6) contains supplementary material, which is available to authorized users.

## Background

Microglial cells are the resident macrophages of the central nervous system (CNS), including the retina, and play a pivotal role in innate immune responses and regulation of homeostasis in the healthy and degenerating CNS [1,2]. Despite being cells of the mononuclear phagocyte lineage, their CNS-specific location and morphology clearly distinguishes them from other macrophage populations [3]. While actively scanning the microenvironment with their long protrusions [4,5], loss of inhibitory signals and the recognition of damage-associated molecular patterns from degenerating neurons lead to the activation of microglia [6-8]. Therefore, reactive microgliosis is a common hallmark of various neurodegenerative diseases including Alzheimer’s disease [9], Parkinson’s disease [10], multiple sclerosis [11], inherited retinal degenerations [12], and several other retinal diseases [13].

We have previously identified activated microglia/macrophage whey acidic protein (AMWAP) as a novel marker of retinal microglial reactivity that is broadly upregulated in several prototypic mouse models of retinal degeneration including retinoschisin-deficient and Fam161a mutant animals [14,15]. AMWAP consists of a 76 aa polypeptide with a cleavable N-terminal 19 aa signal sequence for cellular export and a single 57 aa four-disulfide core domain that is characteristic for all whey acidic proteins [16]. AMWAP overexpression in microglia elicits several immunoregulatory effects including reduction of both pro-inflammatory marker gene expression and migration [14].

The whey acidic protein family is characterized by a highly conserved whey acidic protein domain which is named after the most abundantly expressed protein WAP from rodent milk [17]. AMWAP is closely related to secretory leukocyte protease inhibitor (SLPI), which is the best studied whey acidic protein [18,19]. In contrast to AMWAP, SLPI contains two consecutive WAP domains and is produced at mucosal surfaces as well as by neutrophils and macrophages [20]. SLPI was recently identified as a biomarker for amyotrophic lateral sclerosis [21] and its application has beneficial therapeutic effects after spinal cord injury and optic nerve damage in rodents [22-24].

Toll-like receptor (TLR)-mediated NFκB signaling is a major pathway of pro-inflammatory microglia reactivity that may contribute to chronic neuroinflammation [25,26]. NFκB is tightly regulated via inhibitory κB (IκB) proteins (predominantly IκBα) which mask the nuclear translocation signal of NFκB [27]. TLR ligands including damage-associated molecular patterns from apoptotic retinal neurons [28] and bacterial lipopolysaccharide induce rapid phosphorylation of IκBα by IκB kinase (IKK) followed by ubiquitination and proteasomal degradation within minutes [29,30]. Upon translocation to the nucleus, NFκB rapidly activates the transcription of pro-inflammatory genes. The whey acidic proteins SLPI and elafin both inhibit NFκB activation in monocytes by interfering with degradation of IκBα and IRAK-1, an upstream mediator of NFκB signaling [31,32].

In this study, we used microglial cells and several biochemical and immunological assays to elucidate whether AMWAP also influences NFκB signaling and thereby triggers an anti-inflammatory and neuroprotective phenotype in microglia.

## Methods

### Reagents

*Escherichia coli* 0111:B4 lipopolysaccharide (LPS), zymosan from *Saccharomyces cerevisiae*, Z-Leu-Leu-Leu-al (MG-132), and D-desthiobiotin were purchased from Sigma-Aldrich (St. Louis, MO, USA). CpG oligodeoxynucleotides were purchased from Invivogen (Toulouse, France). N-Acetyl-Leu-Leu-Norleu-al (ALLN) was purchased from Santa Cruz Biotechnology (Dallas, TX, USA). Puromycin dihydrochloride was purchased from Gold Biotechnology (St. Louis, MO, USA).

### Cell culture

BV-2 microglia were cultured in RPMI1640 with 5% fetal calf serum (FCS) supplemented with 2 mM L-glutamine and 195 nM β-mercaptoethanol at 37°C in a humidified atmosphere of 5% CO_2_ as previously described [33]. BV-2 cells were preincubated for 24 h with 10 μg/ml recombinant AMWAP or PBS as vehicle control, unless stated otherwise. BV-2 cells were stimulated with 50 ng/ml LPS, 50 μg/ml zymosan, or 4 μg/ml CpG oligodeoxynucleotides. BV-2 cells were preincubated with the proteasome inhibitor ALLN (100 μg/ml) for 30 min before LPS stimulation to allow for accumulation of phosphorylated proteins. Generation and culture conditions of embryonic stem cell-derived microglia (ESdM) and mouse brain microglia have been described previously [34,35]. ESdM and primary microglia were preincubated for 24 h with 10 μg/ml recombinant AMWAP or PBS as vehicle control and stimulated with 500 ng/ml and 50 ng/ml LPS, respectively. 661W photoreceptor-like cells were a kind gift from Prof. Muayyad Al-Ubaidi (Department of Cell Biology, University of Oklahoma Health Sciences Center, Oklahoma City, OK, USA), and the culture conditions have been described elsewhere [36]. HEK293 EBNA cells were cultured in DMEM low glucose with 1 mM sodium pyruvate and 10% FCS. All media were supplemented with 1% penicillin/streptomycin.

### Recombinant protein expression

Recombinant expression and purification of C-terminally His-tagged AMWAP in *E. coli* was carried out as described previously [14]. For eukaryotic expression of C-terminally Strep(II)-tagged AMWAP, the AMWAP ORF was amplified from BV-2 microglial cDNA with primers forward 5′-cccgctagccacctatgtagtgtcttgccc-3′ and reverse 5′-cccctcgagaaagacaggagttttgcaga-3′ and subcloned into the pCEP-Pu plasmid at restriction sites *Nhe*I and *Xho*I. This plasmid includes the BM-40 signaling peptide which leads to secretion and is then cleaved off from the recombinant protein. The clone was validated by DNA sequencing. HEK293 EBNA cells were transfected with the expression plasmid using the Turbofect™ reagent (Thermo Scientific, Waltham, MA, USA). After 24 h, 3 μg/ml puromycin was added to select for plasmid-positive cells. After expansion of the cells, FCS-free supernatants containing secreted AMWAP were collected every 48 h, centrifuged for 7 min at 3,500 rpm at 4°C and 1 mM PMSF was added. For affinity chromatography, a Strep-Tactin® Sepharose (Iba Life Sciences, Goettingen, Germany) column was prepared and the supernatant loaded onto and passed through the column at a velocity of 0.25 ml/min at 4°C using a peristaltic pump P-1 (GE Healthcare, Little Chalfont, UK). Thereafter, the column was washed with PBS (pH 7.8) and recombinant AMWAP eluted with PBS containing 2.5 mM D-desthiobiotin. Protein-containing fractions were identified using a NanoDrop 2000 (Thermo Scientific), and protein concentration was determined by Bradford assay (Roti®-Quant, Roth, Karlsruhe, Germany) and BCA assay (Roti®-Quant universal, Roth). Recombinant protein was then stored at −20°C until further use.

### Generation of an AMWAP antiserum and Western blot analysis

Rabbit antibodies were raised against an N-terminally GST-tagged recombinant AMWAP peptide. The AMWAP ORF was cloned into a pGEX-4-T1 vector system to express an N-terminally GST-tagged AMWAP protein in prokaryotic cells. The AMWAP ORF was PCR amplified from a plasmid that was described previously [14]. GST-tagged AMWAP protein was expressed and purified as described above. Immunization of rabbits, isolation of serum, and affinity purification was performed by Davids Biotechnologie GmbH (Neutraubling, Germany). For extraction of total cellular protein, BV-2 cells were lysed in cold RIPA buffer (20 mM Na-phosphate buffer, 150 mM NaCl, 5 mM EDTA, 1% Triton X-100, and protease inhibitors) and insoluble debris was removed by centrifugation for 15 min at 13,200 rpm. Nuclear and cytosolic protein extraction was carried out using the NE-PER kit (Thermo Scientific) according to the manufacturer’s instructions. Serum-free microglial supernatants were concentrated 50-fold using Amicon® Ultra-4 centrifugal filter units with a molecular weight cut-off at 3 kDa (Merck-Millipore, Billerica, MA, USA). Protein concentrations were determined by Bradford assay (Roti®-Quant, Roth). A 10 to 20 μg of protein was separated by SDS-PAGE on 10% to 15% gels with PageRuler pre-stained protein ladder (Thermo Scientific). Proteins were then transferred to 0.45 μm nitrocellulose membranes (Bio-Rad, Munich, Germany). After blocking in TBS-T containing 5% nonfat dry milk or bovine serum albumin, membranes were incubated with primary antibodies against AMWAP, NFκB p65 (sc-372, Santa Cruz Biotechnology), phosphorylated NFκB p65 (sc-33020, Santa Cruz Biotechnology), IRAK-1 (sc-7883, Santa Cruz Biotechnology), IκBα (sc-371, Santa Cruz Biotechnology), phosphorylated IκBα (sc-101713, Santa Cruz Biotechnology), ubiquitin (ab7780, Abcam, Cambridge, UK), actin (sc-1616, Santa Cruz Biotechnology), or GAPDH (sc-48166, Santa Cruz Biotechnology). Blots were then incubated with secondary goat anti-rabbit IgG-HRP or rabbit anti-goat IgG-HRP antibodies (sc-2004, sc-2768, Santa Cruz Biotechnology). Enhanced chemiluminescence signals were visualized and imaged with the MultiImage II system (Alpha Innotech, Santa Clara, CA, USA). Total protein visualization was done by Ponceau S staining.

### RNA isolation and reverse transcription

Total RNA was extracted from microglia cells according to the manufacturer’s instructions using the NucleoSpin® RNA Mini Kit (Macherey-Nagel, Dueren, Germany). RNA was quantified spectrophotometrically using a NanoDrop 2000 (Thermo Scientific) and then stored at −80°C. First-strand cDNA synthesis was performed with the RevertAid™ H Minus First strand cDNA Synthesis Kit (Thermo Scientific).

### Quantitative real-time RT-PCR

Amplifications of 50 ng cDNA were performed with an ABI7900HT machine (Applied Biosystems, Carlsbad, CA, USA) in 10 μl reaction mixtures containing 1× TaqMan Universal PCR Master Mix (Applied Biosystems), 200 nM of primers, and 0.125 μl of dual-labeled UPL probe (Roche Applied Science, Basel, Switzerland). The reaction parameters were as follows: 2 min 50°C hold, 30 min 60°C hold, and 5 min 95°C hold, followed by 40 cycles of 20 s 94°C melt and 1 min 60°C anneal/extension. Primer sequences and UPL probe numbers were as follows: IL6, forward primer 5′-gatggatgctaccaaactggat-3′, reverse primer 5′-ccaggtagctatggtactccaga-3′, probe #6; iNOS, forward primer 5′-ctttgccacggacgagac-3′, reverse primer 5′-tcattgtactctgagggctga-3′, probe #13; CCL2, forward primer 5′-catccacgtgttggctca-3′, reverse primer 5′-gatcatcttgctggtgaatgagt-3′, probe #62; CASP11, forward primer 5′-gatcgggcaaccttgaca-3′, reverse primer 5′-tgagattcagttgcttgttgc-3′, probe #72; TNFα, forward primer 5′-ctgtagcccacgtcgtagc-3′, reverse primer 5′-ttgagatccatgccgttg-3′, probe #78; ATP5B, forward primer 5′-ggcacaatgcaggaaagg-3′, reverse primer 5′-tcagcaggcacatagatagcc-3′, probe #77. ATP5B expression was used as the most stable reference gene. Measurements were performed in triplicates, and results were analyzed with the ABI sequence detector software version 2.4 using the ΔΔCt method for relative quantification.

### Fluorescence labeling of recombinant AMWAP

Recombinant AMWAP was fluorescently labeled using the Fluorescein-EX Protein Labeling Kit (Life Technologies, Carlsbad, CA, USA) according to the manufacturer’s instructions. This dye has a seven-atom spacer between the succinimidyl ester group and the fluorophore to minimize unwanted interaction between the fluorophore and the labeled protein. After labeling, the protein solution was passed five times through an Amicon® Ultra-4 centrifugal filter unit with a molecular weight cut-off at 3 kDa (Merck-Millipore) at 4°C and extensively washed with PBS to get rid of excess fluorescent dye.

### Immunocytochemistry

BV-2 cells were seeded on glass cover slips and incubated with 10 μg/ml fluorescently labeled AMWAP or vehicle for different time spans prior to stimulation with 50 ng/ml LPS for 1 h. Cells were then fixed with 4% formaldehyde, washed with PBS, and incubated in blocking buffer containing 10% goat serum and 0.3% Triton X-100. Subsequently, cells were incubated with antibodies against Iba1 or the p65 subunit of NFκB in a solution containing 2.5% goat serum and 0.1% Triton X-100 for 1 h at room temperature. After 30-min incubation with goat anti-rabbit Alexa-594 (A-11012, Life Technologies), slides were washed with PBS and stained with 4′,6-diamidino-2-phenylindole (DAPI). Cover slips were mounted with fluorescent mounting medium (Dako Cytomation, Hamburg, Germany), and fluorescence photomicrographs were taken with an AxioImager.M2 plus ApoTome2 microscope (Carl Zeiss, Oberkochen, Germany). ImageJ software (National Institutes of Health, Bethesda, MD, USA) was used to determine the ratio of nuclear to cytosolic NFκB p65 after quantifying pixel intensities of both cellular compartments and subtracting background fluorescence.

### Immunoprecipitation

In a total volume of 200 μl, cytosolic extracts (200 μg of protein), supplemented with 1× complete protease inhibitor mix (Roche Applied Science), were incubated with 30 μl Protein A-sepharose® 4B (Sigma-Aldrich) for 60 min at 4°C and subsequently centrifuged for 10 min at 2,000 g. The pre-cleared samples were then incubated at 4°C overnight with 2 μg of anti-IκBα antibody (sc-371, Santa Cruz Biotechnology). Thereafter, 30 μl fresh Protein A-sepharose® 4B was added and the mixture was incubated overnight at 4°C to allow for immunoprecipitation. The beads were then pelleted by centrifugation at 2,000 g and the pellet washed five times with PBS. Beads were then incubated in SDS-PAGE sample buffer at 95°C for 5 min and electrophoresed on a 10% SDS-PAGE. The gel was blotted and the membrane incubated with an anti-ubiquitin antibody at 4°C overnight followed by incubation with secondary goat anti-rabbit IgG-HRP at room temperature for 1 h. Enhanced chemiluminescence signals were then visualized and imaged with the MultiImage II system (Alpha Innotech).

### 20S proteasome activity assay

Trypsin-, chymotrypsin-, and caspase-like peptidase activities associated with the 20S proteasome were measured using the Proteasome-Glo™ Assay System (Promega, Madison, WI, USA). A 1 μg/ml of purified 20S proteasome (Enzo Life Sciences, Farmingdale, NY, USA) was incubated at room temperature for 30 min with vehicle, 20 μg/ml AMWAP or 1 μM MG-132 peptidase inhibitor positive control in a 96-well plate containing the substrate for one specific peptidase. Luminescence was then measured with an Infinite F200 Pro plate reader (Tecan, Crailsheim, Germany). A blank reaction without 20S proteasome was used to determine background luminescence associated with the vehicle and Proteasome-Glo™ reagent. The values of the blank reactions were subtracted from all experimental values. Relative luciferase units (RLUs) correspond to the levels of peptidase activity.

### Nitrite measurement

Nitric oxide concentrations were determined by measurement of nitrite released into BV-2 culture supernatants using the Griess reagent system (Promega). Fifty microliter cell culture supernatants from control, 50 ng/ml LPS-, 10 μg/ml AMWAP-, or 50 ng/ml LPS + 10 μg/ml AMWAP-treated BV-2 microglia were incubated with 100 μl Griess reagent in each well of a translucent 96-well plate. After incubation for 30 min at room temperature, absorbance was measured at 540 nm on an Infinite F200 Pro plate reader (Tecan). Nitrite concentrations were calculated on the basis of a sodium nitrite reference curve.

### 661W photoreceptor apoptosis assay

To investigate microglial neurotoxicity, 661W photoreceptor cells were incubated for 48 h with culture supernatants from control, 50 ng/ml LPS-, 10 μg/ml AMWAP-, or 50 ng/ml LPS + 10 μg/ml AMWAP-treated BV-2 microglia. Apoptotic cell death was determined using the Caspase-Glo® 3/7 Assay (Promega). Cells were lysed and incubated with a luminogenic caspase-3/7 substrate, which contains the tetrapeptide sequence DEVD. After incubation at room temperature for 1 h, the generated luminescence was measured on an Infinite F200 Pro plate reader (Tecan). A blank reaction without cells was used to determine background luminescence associated with the cell culture system and Caspase-Glo® 3/7 reagent. The values of the blank reactions were subtracted from all experimental values. Negative control reactions were performed to determine the basal caspase activity of 661W cells. Relative luciferase units (RLUs) reflect the level of apoptotic cell death.

### Phalloidin-TRITC staining

BV-2 cells were seeded on cover slips in six-well plates, and attachment was allowed overnight. Cells were then preincubated with 10 μg/ml AMWAP or vehicle for 24 h before incubation with 50 ng/ml LPS for 24 h. Thereafter, cells were fixed with 4% formaldehyde, permeabilized with 0.1% Triton X-100, and F-actin was fluorescently labeled using 0.1 μg/ml Phalloidin-TRITC (Sigma-Aldrich). Nuclei were stained using DAPI, and the cover slips were mounted with fluorescent mounting medium (Dako Cytomation). Photomicrographs were taken with an AxioImager.M2 plus ApoTome2 microscope (Carl Zeiss).

### Proliferation assay

For carboxyfluorescein diacetate succinimidyl ester (CFSE) proliferation assay, BV-2 microglia were labeled with 5 μM CFSE (e-Bioscience, San Diego, CA, USA) and cultured in a six-well plate. After 24 h of AMWAP or vehicle pretreatment and subsequent 24 h of LPS treatment, cells were stained with a fixable viability dye (e-Bioscience) to exclude dead cells from the analysis. The fluorescence intensity of CFSE-labeled BV-2 cells was analyzed by flow cytometry on a FACS Canto II (BD Biosciences, San Jose, CA, USA). Analysis of cell division rate was performed using FlowJo software (Treestar Inc., Ashland, OR, USA).

### Phagocytosis assay

661W photoreceptor cells were starved by serum deprivation and harvested and fluorescently labeled using CellTracker CM-DiI (Invitrogen, Carlsbad, CA, USA). For phagocytosis assay, BV-2 cells were seeded on cover slips in six-well plates and attachment was allowed overnight. After pretreatment with 10 μg/ml AMWAP for 24 h and subsequent stimulation with 50 ng/ml LPS for 24 h, 500 μl-stained apoptotic 661W cell suspension was added for 6 h. After extensive washing with PBS to get rid of remaining extracellular 661W debris, cells were fixed and nuclei were stained with DAPI. Fluorescence micrographs with constant exposure times were taken with an AxioImager.M2 plus ApoTome2 microscope (Carl Zeiss). ImageJ software (National Institutes of Health) was used to determine the ratio of phagocytosed apoptotic photoreceptor fragments (background-corrected red signal) relative to the total microglia cell number (background-corrected DAPI signal).

### Statistical analysis

Quantitative real-time RT-PCR data were analyzed with the ΔΔCt method using an unpaired Student’s *t*-test. Assays for NFκB localization, 20S proteasome inhibition, nitrite secretion, caspase 3/7, and phagocytic activity were analyzed with a Mann–Whitney U-test. *P* < 0.05 was considered statistically significant.

## Results

### AMWAP is secreted from reactive microglia

**Figure 1:**
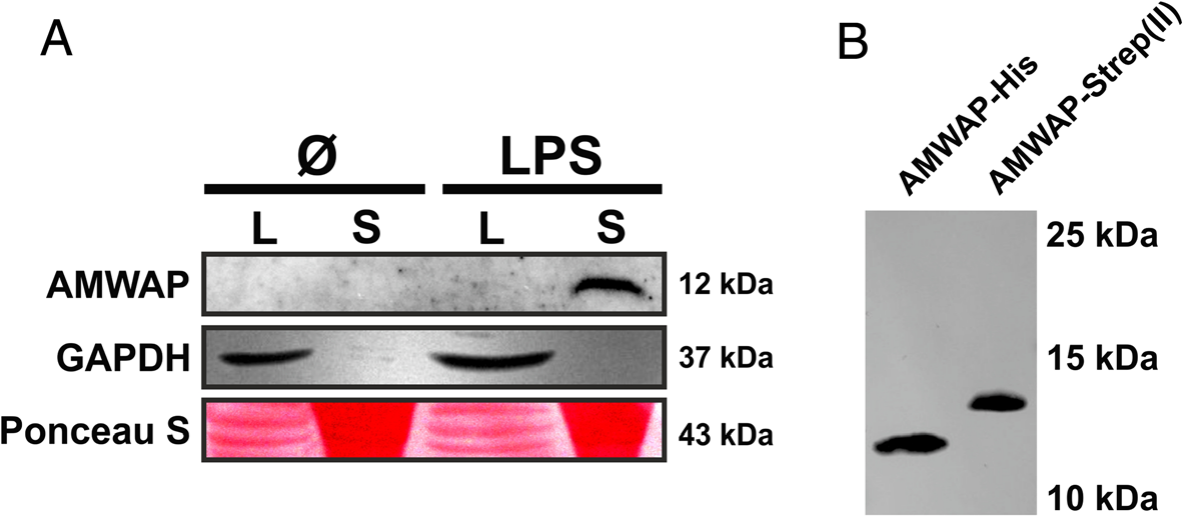
AMWAP is secreted from reactive BV-2 microglia. **(A)** BV-2 microglial cells were treated with 50 ng/ml LPS for 48 h before obtaining cellular protein lysates (L) as well as concentrated culture supernatants (S). Anti-AMWAP immunoblot analysis revealed a band of approximately 12 kDa only present in the supernatant of LPS-activated microglia. GAPDH and Ponceau S served as loading controls. **(B)** Anti-AMWAP immunoblot analysis of recombinantly expressed and purified AMWAP from *E. coli* (AMWAP-His) and HEK293 EBNA cells (AMWAP-Strep(II)) shows single bands at approximately 11 to 13 kDa.

To elucidate whether AMWAP is secreted from reactive microglia, we treated BV-2 microglial cells with 50 ng/ml LPS for 48 h and analyzed AMWAP in cell culture supernatants and cellular extracts using a newly generated anti-AMWAP antibody. The Western blot analysis detected a single specific band at approximately 12 kDa in culture supernatants of LPS-activated BV-2 microglia but not in unstimulated cells (Figure 1A). Anti-GAPDH immunoblotting and Ponceau S staining served as loading controls for intracellular and secreted proteins. Thus, we conclude that AMWAP can be actively secreted from microglia when stimulated with a pro-inflammatory trigger such as the TLR4 ligand LPS.

To analyze the paracrine effects of AMWAP, we next carried out recombinant expression of C-terminally His-tagged and C-terminally Strep(II)-tagged AMWAP using *E. coli* and HEK293 EBNA cells, respectively. After purification by affinity chromatography, recombinant proteins were immunoblotted and detected with the anti-AMWAP antibody. Specific single bands were present at approximately 11 kDa for AMWAP-His and approximately 13 kDa for AMWAP-Strep(II) (Figure 1B). These recombinant AMWAP proteins were then used for all further functional studies.

### AMWAP reduces the TLR-mediated pro-inflammatory microglia response through NFκB inhibition

**Figure 2:**
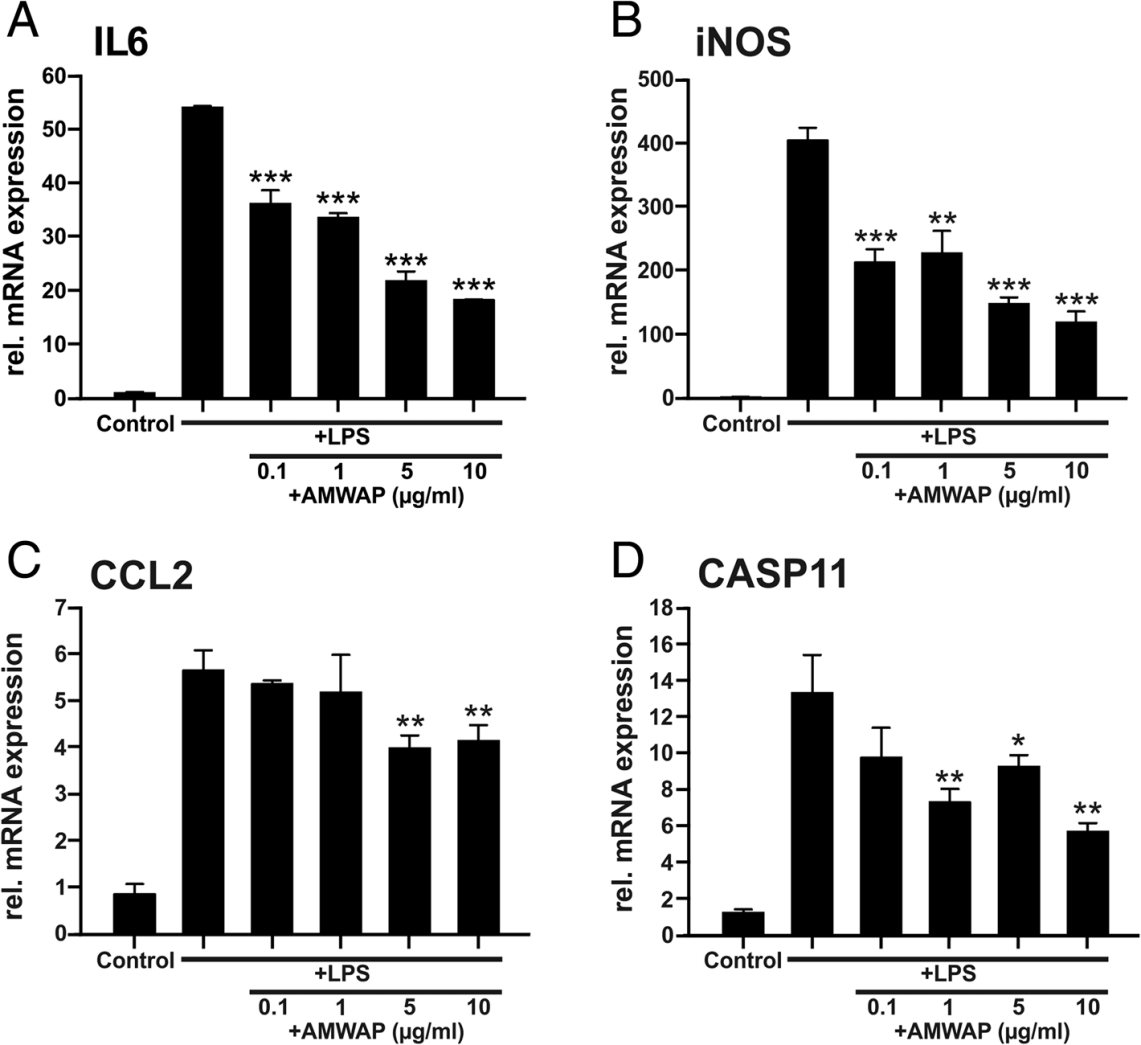
AMWAP reduces pro-inflammatory marker gene transcription in BV-2 microglia. BV-2 cells were treated with various concentrations of recombinant AMWAP for 24 h before further stimulation with 50 ng/ml LPS for 12 h. Transcript levels of the pro-inflammatory markers IL6 **(A)**, iNOS **(B)**, CCL2 **(C),** and CASP11 **(D)** were determined by quantitative real-time PCR. Data show mean ± SD (*n* = 3/group, measured in triplicate) with *P < 0.05, **P < 0.01, ***P < 0.001 for AMWAP + LPS- *vs.* LPS-treated cells. PBS served as vehicle control.

Next, we investigated whether exogenous stimulation with AMWAP influences pro-inflammatory microglia reactivity in BV-2 cells. We tested the effects of different AMWAP concentrations ranging from 0.1 to 10 μg/ml on TLR4/LPS-induced mRNA expression of the pro-inflammatory mediators IL6, iNOS, CCL2, and CASP11 using quantitative real-time RT-PCR. These specific transcripts were selected as surrogate markers for the key microglia pathways pro-inflammatory cytokines, radical production, chemotaxis, and inflammatory caspases. AMWAP strongly and dose-dependently suppressed mRNA levels of IL6 and iNOS and had a less pronounced but significant effect on CCL2 and CASP11 gene expression (Figure 2).

**Figure 3:**
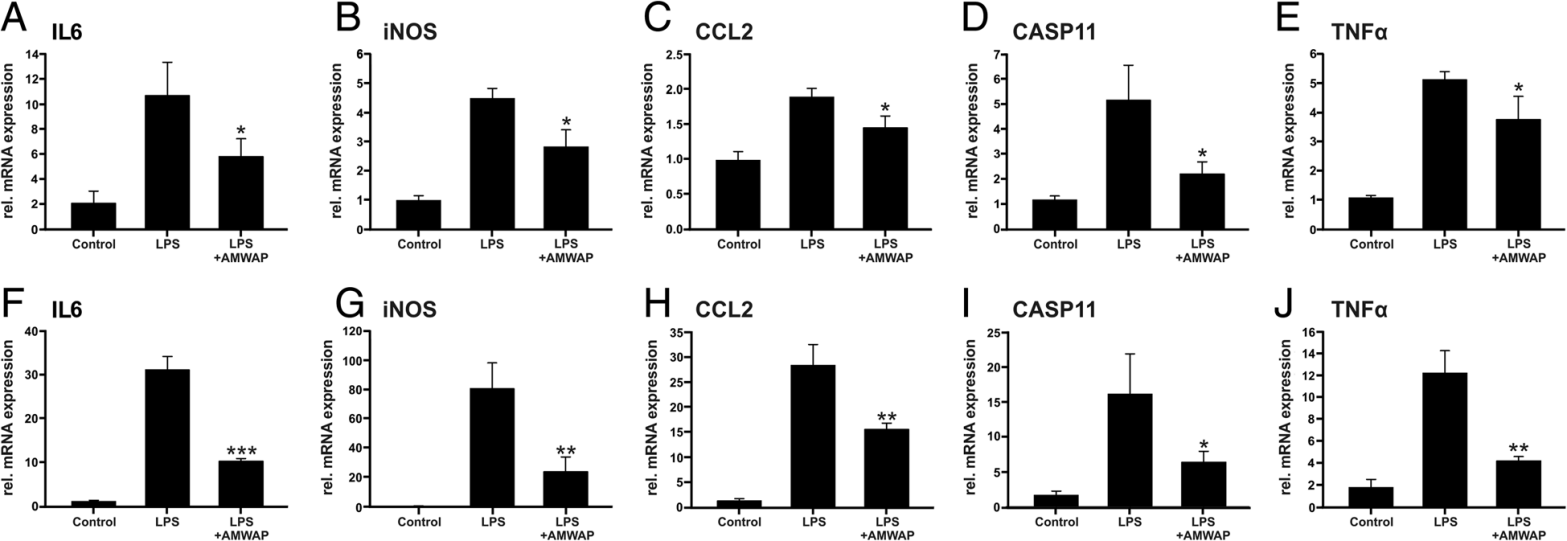
AMWAP reduces TLR4-mediated pro-inflammatory gene transcription in embryonic stem cell-derived microglia and primary brain microglia. **(A-E)** Embryonic stem cell-derived microglia (ESdM) were treated with 10 μg/ml of recombinant AMWAP for 24 h before stimulation with 500 ng/ml LPS for further 24 h. **(F-J)** Primary brain microglia were treated with 10 μg/ml of recombinant AMWAP for 24 h before stimulation with 50 ng/ml LPS for further 24 h. mRNA expression of the pro-inflammatory marker transcripts IL6 (A, F), iNOS (B, G) CCL2 (C, H), CASP11 (D, I), and TNFα (E, J) was determined by quantitative real-time RT-PCR. Data show mean ± SD (*n* = 3/group, measured in triplicate) with *P < 0.05, **P < 0.01, ***P < 0.001 for AMWAP + LPS- *vs*. LPS-treated cells. PBS served as vehicle control.

Since BV-2 is a microglial cell line, we then validated these findings in embryonic stem cell-derived microglia (ESdM), a recently established model system which exhibits many properties of primary microglia [35,37]. ESdM pretreated with 10 μg/ml AMWAP showed significantly reduced mRNA expression of the pro-inflammatory mediators IL6, iNOS, CCL2, CASP11, and TNFα after LPS activation (Figure 3A-E). These results were also fully replicated in primary mouse brain microglia (Figure 3F-J), indicating that BV-2 microglia are a suitable model to study the effects of AMWAP on microglia.

**Figure 4:**
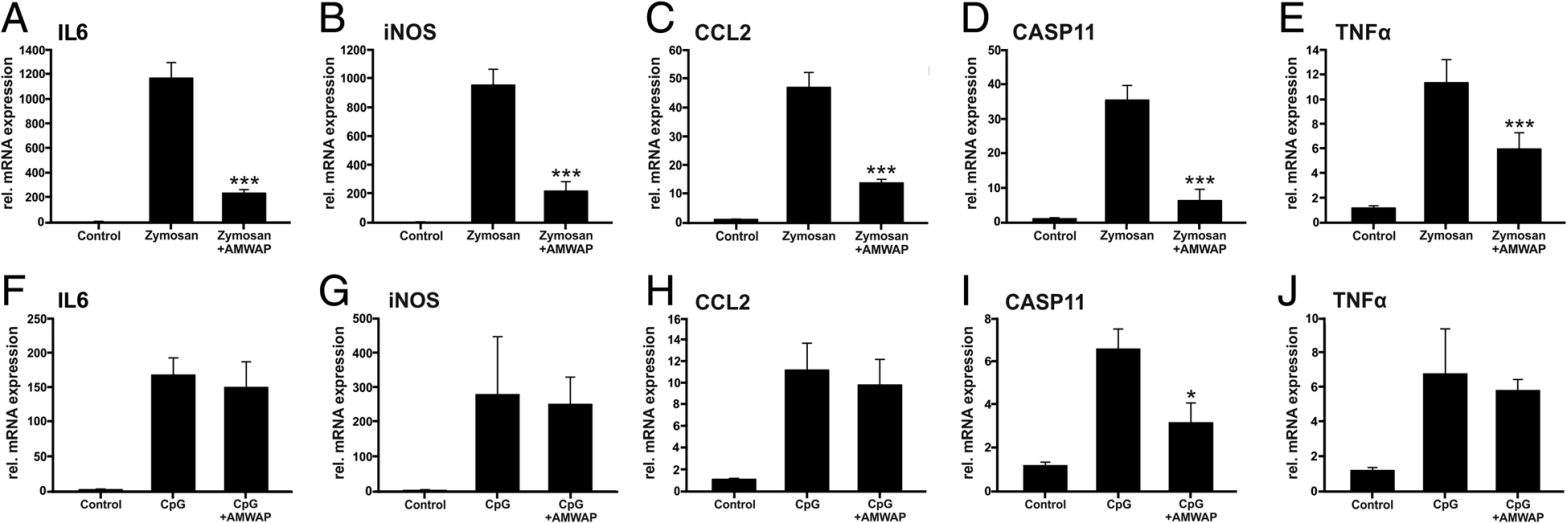
AMWAP reduces TLR2-mediated pro-inflammatory gene transcription in BV-2 microglia. BV-2 cells were treated with 10 μg/ml of recombinant AMWAP for 24 h before stimulation with 50 μg/ml zymosan **(A-E)** or 4 μg/ml CpG oligodeoxynucleotides **(F-J)** for additional 24 h. The pro-inflammatory marker transcripts IL6 (A, F), iNOS (B, G), CCL2 (C, H), CASP11 (D, I), and TNFα (E, J) were measured by quantitative real-time RT-PCR. Data show mean ± SD (*n* = 3/group, measured in triplicate) with *P < 0.05, **P < 0.01, ***P < 0.001 for AMWAP + zymosan/CpG- *vs*. zymosan/CpG-treated cells. PBS served as vehicle control.

We next investigated a potential anti-inflammatory effect of AMWAP on microglial cells that were activated with TLR2 and TLR9 ligands, respectively. TLR2, as does TLR4, acts on the cell surface and predominantly signals via NFκB, whereas intracellular TLR9 activation mainly stimulates interferon regulatory factors and has only a minor effect on NFκB targets [38]. BV-2 microglia pretreated with 10 μg/ml AMWAP showed strongly reduced mRNA expression of IL6, iNOS, CCL2, CASP11, and TNFα after activation with the TLR2 ligand zymosan (Figure 4A-E). In contrast, AMWAP only weakly influenced gene transcription of CASP11 triggered by CpG oligodeoxynucleotides as TLR9 ligands (Figure 4F-J).

**Figure 5:**
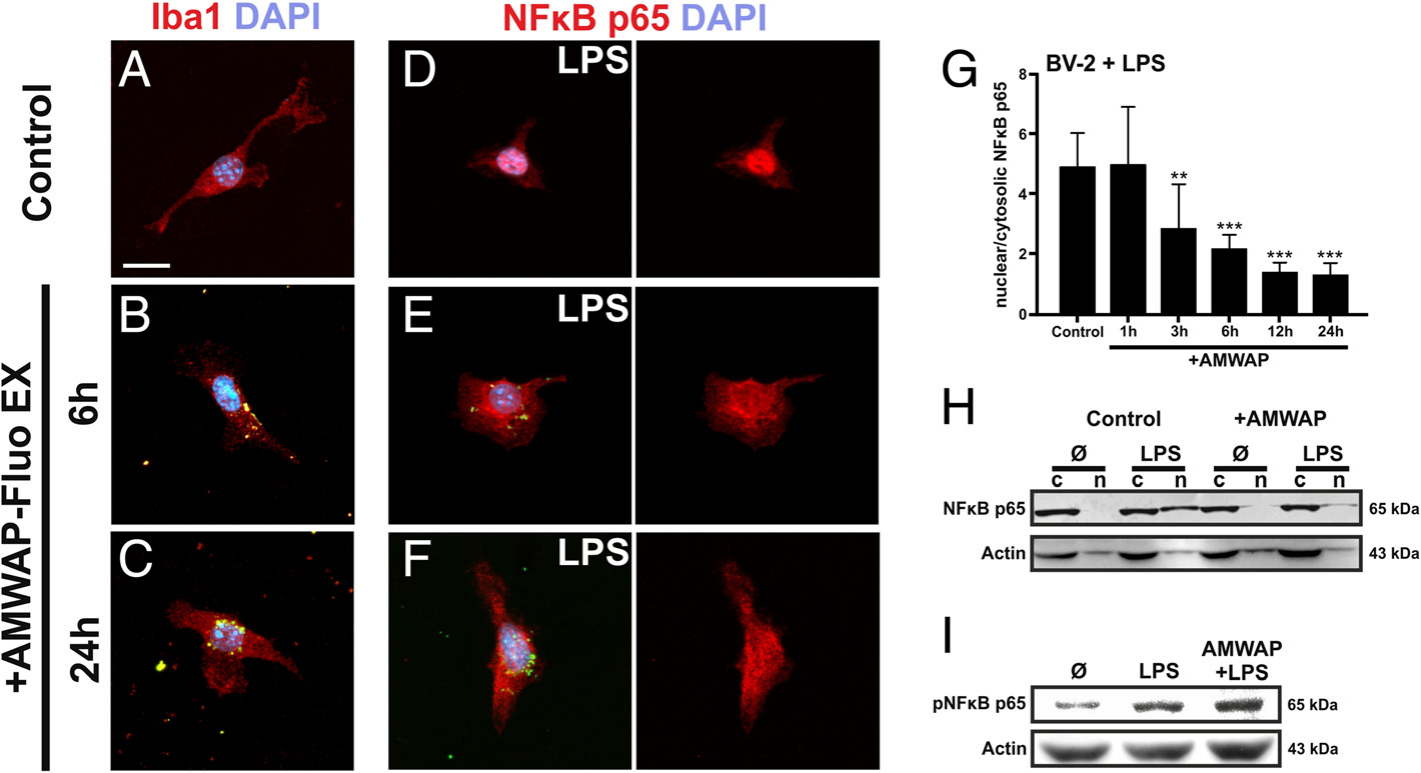
AMWAP is taken up by microglia and inhibits LPS-mediated NFκB activation. **(A-C)** BV-2 microglia were incubated with fluorescently labeled recombinant AMWAP (AMWAP-Fluo EX, 10 μg/ml) for 6 and 24 h. Microglia gradually incorporated AMWAP-Fluo EX into their cytosol exhibiting a perinuclear localization as shown by anti-Iba1 antibody co-staining. **(D-G)** AMWAP-Fluo EX pretreatment (for 1 to 24 h) time-dependently inhibited TLR4-mediated nuclear translocation of NFκB p65 after stimulation with 50 ng/ml LPS for 1 h as shown by immunocytochemistry and quantification of nuclear *vs.* cytosolic fluorescence intensities. PBS served as vehicle control. Data show mean ± SD (*n* = 9/group) with *P < 0.05, **P < 0.01, ***P < 0.001 for AMWAP-treated *vs.* control cells. **(H)** Control and AMWAP-treated BV-2 microglia were stimulated with 50 ng/ml LPS for 1 h and NFκB p65 protein amount was determined in cytosolic (*c*) and nuclear (*n*) fractions using Western blot. AMWAP-treated cells exhibited diminished LPS-triggered NFκB p65 translocation to the nucleus compared to vehicle control. **(I)** Control and AMWAP-treated BV-2 microglia were stimulated with 50 ng/ml LPS for 1 h and the NFκB p65 phosphorylation status (pNFκB p65) was determined in total cell lysates using Western blot. Actin served as loading control. Scale bar = 20 μm.

To investigate whether exogenous AMWAP is taken up by microglia and potentially influences NFκB activation, we treated BV-2 cells with 10 μg/ml fluorescently labeled AMWAP (AMWAP-Fluo EX) for different time periods. AMWAP-Fluo EX was gradually incorporated by microglia and ultimately localized to the cytosol in a perinuclear fashion (Figure 5A-C). Upon stimulation with 50 ng/ml LPS for 1 h, TLR4-mediated NFκB p65 translocation to the nucleus was effectively inhibited in AMWAP-positive cells as shown by quantitative immunocytochemistry (Figure 5D-G). These findings were confirmed by Western blot analysis of cytosolic *vs.* nuclear NFκB p65 levels for control and AMWAP-treated BV-2 microglia with or without stimulation with 50 ng/ml LPS for 1 h (Figure 5H). AMWAP did not interfere with LPS-induced phosphorylation of NFκB p65 in BV-2 microglia (Figure 5I), indicating that AMWAP acts mainly through inhibition of nuclear NFκB p65 translocation rather than blocking NFκB p65 phosphorylation. These data are in agreement with observations from microglia overexpressing AMWAP-GFP, which showed diminished nuclear NFκB p65 translocation upon LPS-stimulation (Additional file 1: Figure S1).

### AMWAP prevents LPS-triggered degradation of IRAK-1 and IκBα

**Figure 6:**
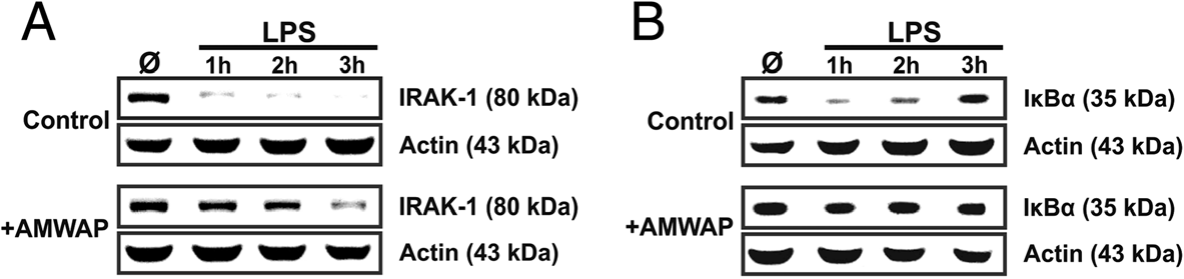
AMWAP prevents LPS-induced degradation of IRAK-1 and IκBα. Control and AMWAP-treated BV-2 microglia were incubated with 50 ng/ml LPS for 0, 1, 2, and 3 h, and cytosolic protein extracts were prepared. Immunoblot analysis of IRAK-1 **(A)** and IκBα **(B)** was carried out to determine the level of proteolytic degradation. In AMWAP-treated cells, both signaling molecules were protected from LPS-induced proteolysis. Resynthesized IκBα was detected after 2 and 3 h in control cells. Actin served as loading control.

To further investigate the mechanisms of AMWAP on microglial NFκB signaling, we analyzed the protein levels of the NFκB pathway mediators IRAK-1 and IκBα in a short time course of LPS and AMWAP co-treatment. Western blot analysis of cytosolic fractions showed a rapid degradation of both signaling proteins as early as 1 h after LPS stimulation of microglia (Figure 6), with apparent IκBα resynthesis starting after 2 h (Figure 6B). Treatment with AMWAP effectively protected IRAK-1 and particularly IκBα from rapid LPS-induced proteolysis.

### AMWAP acts independent of IκBα phosphorylation, ubiquitination, or the 20S proteasome

**Figure 7:**
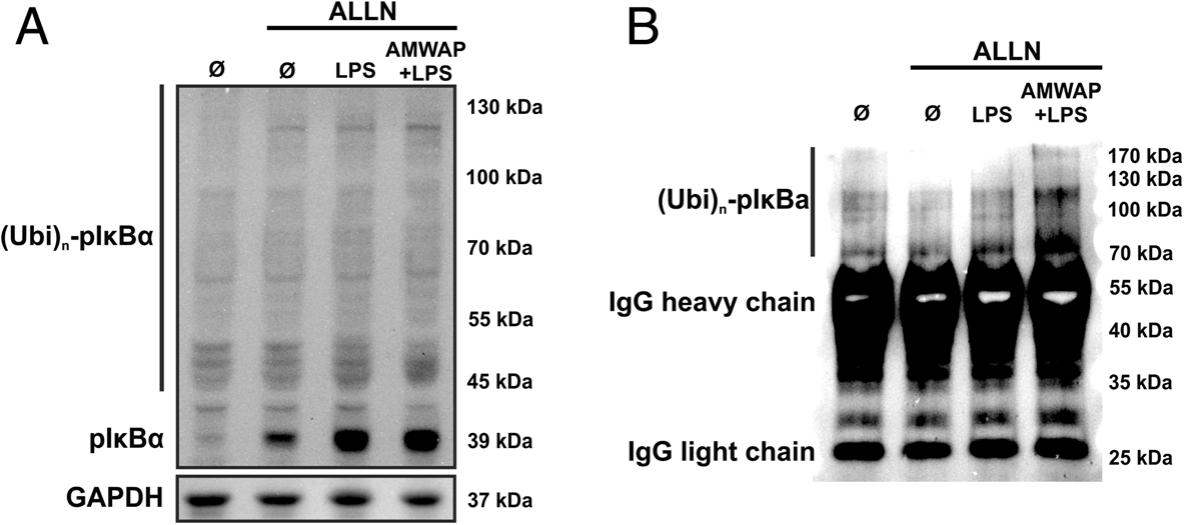
AMWAP does not inhibit IκBα phosphorylation and ubiquitination. **(A)** Control and AMWAP-treated BV-2 microglia were preincubated with the proteasome inhibitor ALLN (100 μg/ml) for 30 min to allow for accumulation of phosphorylated IκBα before stimulation with LPS (50 ng/ml) for 30 min. Levels of phosphorylated IκBα were assessed in cytoplasmic extracts using Western blot analysis. AMWAP did not reduce LPS-induced IκBα phosphorylation. The presence of various high molecular weight bands was noticed, presumably representing polyubiquitinated, phosphorylated IκBα. **(B)** IκBα was immunoprecipitated from cytoplasmic samples of cells treated as in (A). Thereafter, anti-ubiquitin immunoblot revealed the presence of high molecular weight polyubiquitinated forms of phosphorylated IκBα, which were not diminished in AMWAP-positive cells. GAPDH served as loading control. (p)IκBα, (phosphorylated) inhibitor of kappa B alpha; Ubi, ubiquitin; IgG, immunoglobulin G; ALLN; N-acetyl-Leu-Leu-Norleu-al.

We next assessed the effect of AMWAP on LPS-induced phosphorylation of IκBα. IκBα phosphorylation is an upstream event of NFκB p65 nuclear translocation and thus a critical event in NFκB signaling. BV-2 microglia were preincubated with the proteasome inhibitor ALLN (100 μg/ml) for 30 min to allow for accumulation of labile phosphorylated IκBα species. After stimulation with 50 ng/ml LPS for 30 min, the amount of phosphorylated IκBα in cytosolic extracts was analyzed using Western blot. Phosphorylated IκBα levels were significantly elevated in LPS-treated cells (Figure 7A). However, this rapid IκBα phosphorylation was not changed in AMWAP-treated cells (Figure 7A). In this Western blot, we noticed the presence of multiple high molecular weight bands, which presumably represent polyubiquitinated, phosphorylated IκBα-species. To verify this assumption, we immunoprecipitated IκBα from the cytoplasmic samples and carried out anti-ubiquitin immunoblot analysis. This experiment clearly revealed the presence of high molecular weight polyubiquitinated forms of phosphorylated IκBα between 70 and 130 kDa in LPS-treated microglia, which were not reduced by AMWAP treatment (Figure 7B).

**Figure 8:**
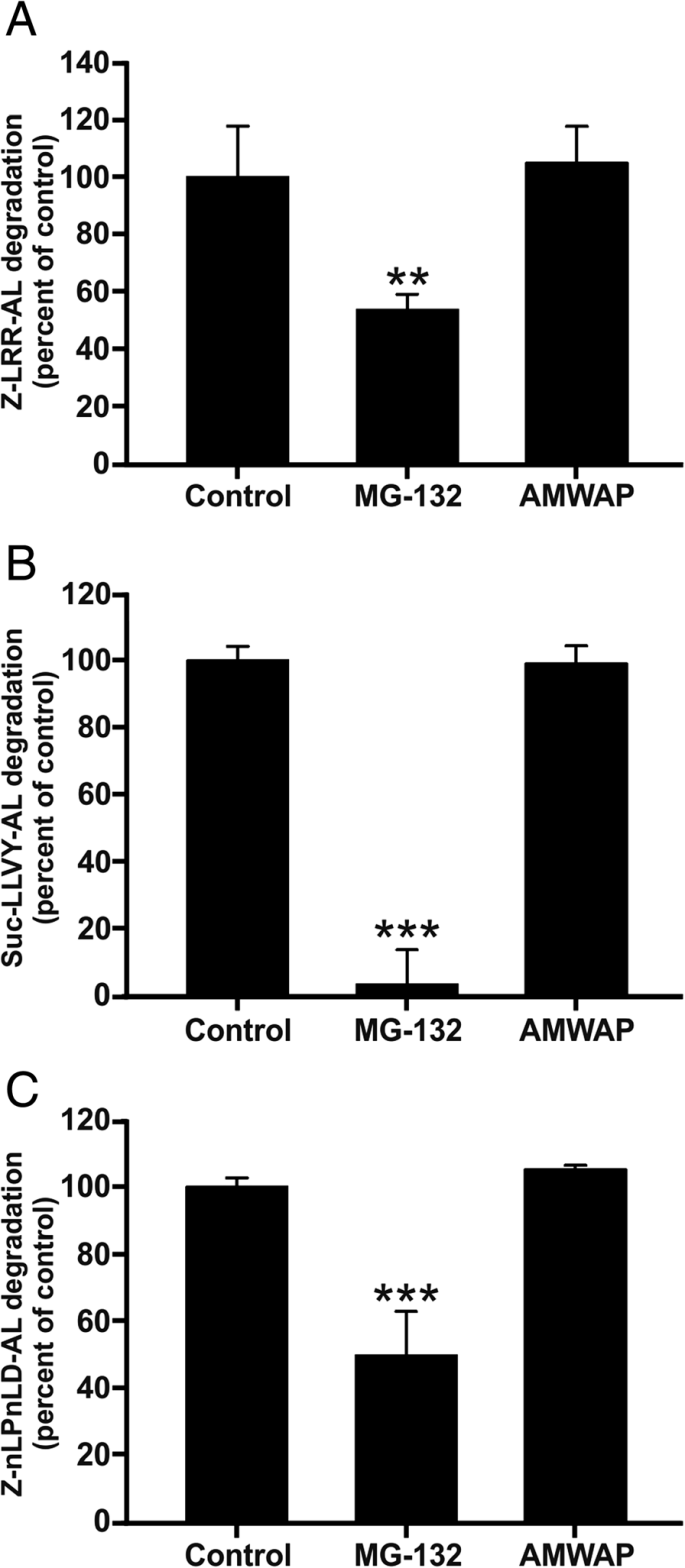
AMWAP does not inhibit peptidase activities of the 20S proteasome. The effect of recombinant AMWAP (20 μg/ml) on peptidase activities of the 20S proteasome was assayed using luminogenic cleavage assays with the substrates Z-LRR-aminoluciferin (trypsin-like activity) **(A)**, Suc-LLVY-aminoluciferin (chymotrypsin-like activity) **(B)**, and Z-nLPnLD-aminoluciferin (caspase-like activity) **(C)**. Data show mean ± SD (*n* = 4/group) with *P < 0.05, **P < 0.01, ***P < 0.001 for treatment *vs.* control. MG-132 (1 μM), a potent 20S proteasome inhibitor, served as a positive control that reduced all three peptidase activities. PBS served as a vehicle control. MG-132, Z-Leu-Leu-Leu-al.

Of note, we observed that AMWAP-treated BV-2 microglia accumulated phosphorylated IκBα after stimulation with 50 ng/ml LPS for 30 min even in the absence of the proteasome inhibitor ALLN and even slightly inhibited the basal turnover of (phosphorylated) IκBα under non-LPS-treated conditions (Additional file 2: Figure S2). These findings prompted us to investigate whether AMWAP reduces IκBα proteolysis due to direct inhibition of the 20S proteasome. A luminogenic cleavage assay using the substrates Z-LRR-aminoluciferin (for trypsin-like activity), Suc-LLVY-aminoluciferin (for chymotrypsin-like activity), and Z-nLPnLD-aminoluciferin (for caspase-like activity) was used to test the three different 20S peptidase activities. There was no influence on 20S peptidase activities by AMWAP in contrast to a strong reduction of all three activities by the proteasome inhibitor MG-132 (Figure 8). Based on these findings, we conclude that the AMWAP-triggered inhibition of IκBα degradation is not mediated by direct interference with 20S proteasome activity.

### AMWAP induces a neuroprotective microglia phenotype

**Figure 9:**
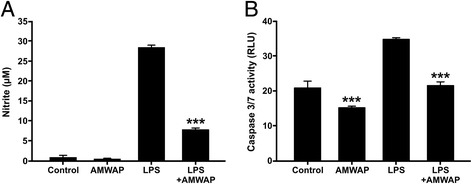
AMWAP reduces pro-inflammatory microglial nitric oxide production and neurotoxicity on photoreceptor cells. **(A)** Production of nitric oxide (NO) as determined by detection of nitrite from BV-2 microglial cells pre-treated with 10 μg/ml AMWAP or vehicle for 24 h before stimulation with 50 ng/ml LPS for further 24 h. Data show mean ± SD (*n* = 6/group) with *P < 0.05, **P < 0.01, ***P < 0.001 for AMWAP + LPS *vs.* LPS-treated cells. **(B)** 661W photoreceptor cells were incubated with conditioned media from vehicle-, 10 μg/ml AMWAP-, 50 ng/ml LPS-, and 50 ng/ml LPS + 10 μg/ml AMWAP-treated BV-2 microglia for 48 h and apoptosis-related caspase 3/7 activity was determined. Data show mean ± SD (*n* = 5/group) with *P < 0.05, **P < 0.01, ***P < 0.001 for AMWAP-treated cells *vs.* control and AMWAP + LPS *vs.* LPS-treated cells, respectively. PBS served as a vehicle control. NO, nitric oxide; RLU, relative luciferase units.

We next aimed to investigate the effects of recombinant AMWAP on pro-inflammatory microglial functions including the production of toxic oxygen radicals and direct effects on retinal neurons. As AMWAP significantly reduced iNOS gene expression in LPS-treated microglia (Figure 2A, Figure 3B,G), we also tested its influence on NO production. AMWAP effectively diminished the strong LPS-induced NO secretion into the microglia culture supernatant (Figure 9A). To further test the influence of AMWAP on microglial neurotoxicity, 661W photoreceptor cells were incubated for 48 h with conditioned media from BV-2 cells and caspase-related apoptotic cell death was monitored. 661W photoreceptors cultured in the presence of microglial supernatants derived from LPS-activated cells showed higher caspase 3/7 activity, which was effectively reduced when microglia were treated with AMWAP (Figure 9B). These data suggest that AMWAP limits microglial production of neurotoxic molecules and may potentially induce the release of neurotrophic factors.

**Figure 10:**
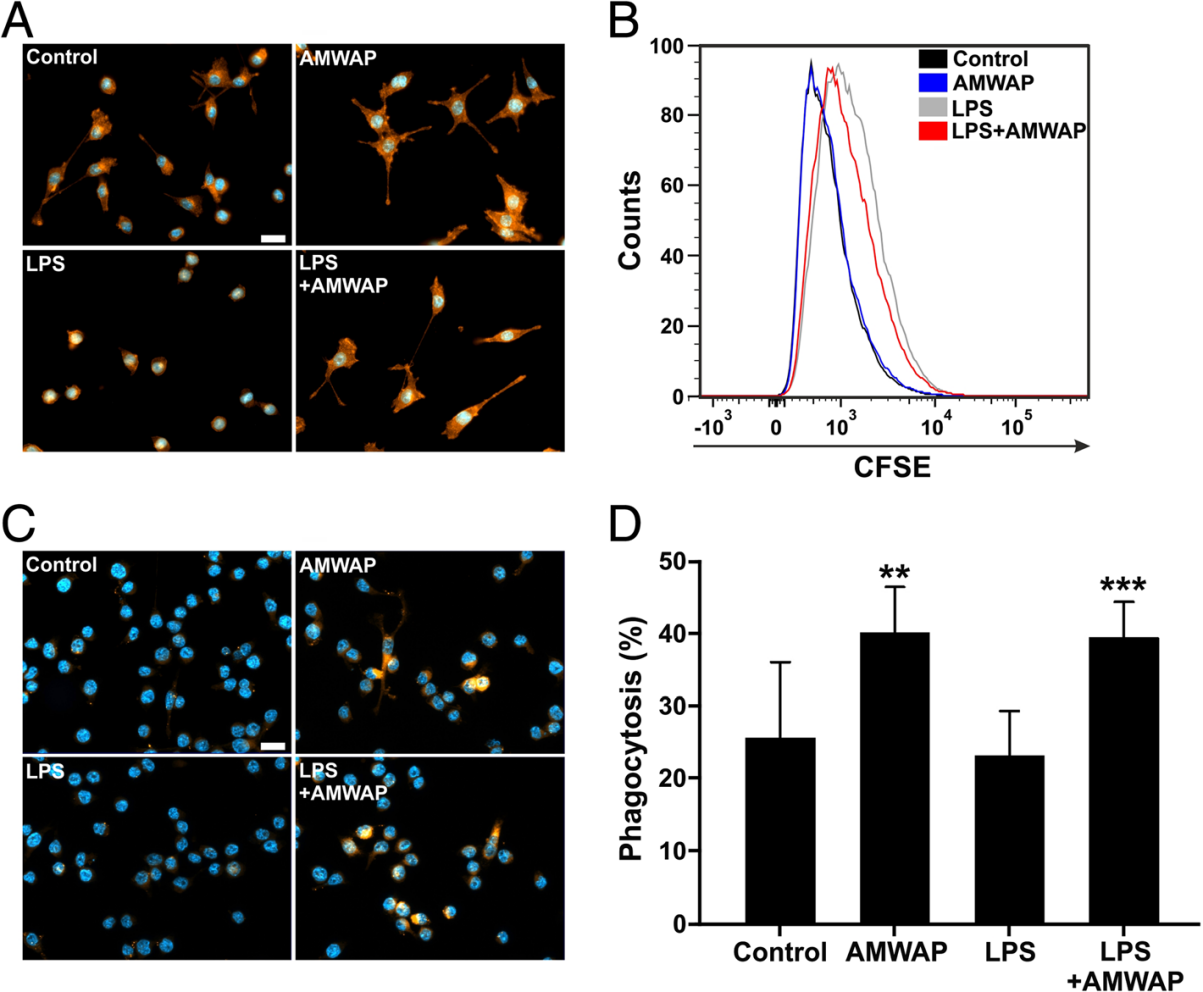
AMWAP promotes microglial filopodia formation and phagocytosis. **(A)** Representative images of Phalloidin-TRITC labeled BV-2 microglial cells pre-treated with 10 μg/ml AMWAP or vehicle for 24 h in the absence or presence of 50 ng/ml LPS for 24 h. **(B)** CFSE-proliferation assay of BV-2 microglia treated with 10 μg/ml AMWAP or vehicle for 24 h in the absence or presence of 50 ng/ml LPS for 24 h. The proliferation rate was assessed 24 h after LPS treatment using flow cytometry and a representative graph out of three repetitions is shown. **(C, D)** Phagocytosis of CM-DiI-stained apoptotic 661W photoreceptor debris by BV-2 microglia was monitored and quantified after 6-h feeding time of AMWAP- *vs.* vehicle-treated cells in the absence or presence of 50 ng/ml LPS for 24 h. Data show mean ± SD (*n* = 9/group) with *P < 0.05, **P < 0.01, ***P < 0.001 for AMWAP-treated cells *vs.* control and AMWAP + LPS *vs.* LPS-treated cells, respectively. PBS served as a vehicle control. CFSE, carboxyfluorescein diacetate succinimidyl ester. Scale bar = 20 μm.

As filopodia formation is a hallmark of homeostatic microglia, we investigated the effect of AMWAP on microglia morphology. LPS-activated or control BV-2 cells were cultured in the absence or presence of AMWAP, and their F-actin cytoskeleton was stained using Phalloidin-TRITC. AMWAP treatment induced filopodia formation in control microglia and even reversed the amoeboid phenotype observed after LPS activation (Figure 10A). CFSE labeling and FACS analysis demonstrated that AMWAP did not exert significant anti-mitotic effects on BV-2 cells, which could have been a potential bias in cell shape (Figure 10B). We finally analyzed phagocytosis of cellular debris as another parameter of the non-inflammatory housekeeping function of microglia. For this purpose, CM-DiI-stained apoptotic 661W photoreceptor fragments were exposed to control and LPS-treated cells in the absence or presence of AMWAP and the amount of phagocytosed debris was monitored. AMWAP significantly increased the phagocytic uptake of 661W photoreceptor debris in the presence or absence of LPS, indicating its important regulatory activity on microglia (Figure 10C,D).

## Discussion

We have previously shown that AMWAP overexpression in microglia induces an anti-inflammatory counter-regulatory phenotype and that knockdown of AMWAP leads to higher intrinsic pro-inflammatory cytokine expression [14]. AMWAP expression and secretion are themselves controlled by NFκB as blockade of NFκB activation inhibits LPS-mediated induction of AMWAP expression [14]. In the present study, we show that AMWAP is a secreted protein of reactive microglia that acts in a paracrine fashion to prevent TLR4- and TLR2-triggered NFκB translocation. Furthermore, we demonstrate here that AMWAP interferes with the proteolytic degradation of the regulator proteins IRAK-1 and IκBα. This function of AMWAP in microglia is reminiscent of SLPI, another member of the WAP family, which is secreted from macrophages and acts as a suppressor of LPS responses [39].

To obtain larger amounts of recombinant protein for further exploration of the immunoregulatory role of AMWAP, we first generated prokaryotic AMWAP. Since previous studies on SLPI function demonstrated improved folding and disulfide bond formation when expressed in the yeast *Pichia pastoris* [40,41], we then also obtained AMWAP from a eukaryotic source. Despite the fact that both recombinant AMWAP forms used in our study exerted similar NFκB inhibitory and anti-inflammatory functions, the risk of LPS contamination is significantly reduced when using eukaryotic HEK293 EBNA cells for AMWAP expression.

We showed that AMWAP dampened the TLR4-induced expression of the bona fide NFκB target genes IL6, iNOS, CCL2, CASP11, and TNFα in BV-2 microglia, as well as in ESdM cells, which closely mimic primary microglia regarding their transcriptomic profile [37] and in mouse brain microglia. We also found that AMWAP effectively reduced TLR2-mediated pro-inflammatory gene expression but had only weak effects on TLR9 signaling. TLR2 signals mainly through NFκB, while TLR9 acts predominantly via interferon regulatory factors (IRFs) [38,42]. Therefore, a plausible explanation for the lack of AMWAP-mediated inhibition of IL6, iNOS, CCL2, and TNFα after CpG oligodeoxynucleotide treatment is that these genes are also IRF regulated [43-46] whereas TLR-induced CASP11 expression is mediated by NFκB and independent from IRFs [47]. These findings are also consistent with data from other whey acidic proteins as SLPI has been reported to inhibit TLR2- and TLR4-mediated NFκB activation in human monocytic U937 cells [48].

Together with our previous observation that AMWAP inhibits NFκB-dependent CCL2 promoter activity [14], these findings prompted us to study the direct influence of AMWAP on microglial NFκB activation. Exogenous AMWAP was taken up by microglia and effectively inhibited LPS-triggered nuclear NFκB p65 translocation. This effect very likely works in an autocrine and paracrine way as AMWAP is actively secreted from pro-inflammatory microglia. Upon LPS stimulation, NFκB p65 is rapidly phosphorylated at Ser536 by IκB kinases (IKKs) in the cytosol, increasing its transactivation potential once translocated into the nucleus [49]. We did not observe an inhibition of NFκB p65 phosphorylation through AMWAP, indicating that the anti-inflammatory effect of AMWAP is mainly due to cytosolic NFκB retention. We observed a ‘patchy’ perinuclear pattern of fluorescently labeled AMWAP in the cells, which raised the question of a possible localization in subcellular compartments. Interestingly, SLPI is located in cellular granules in neutrophils and can be released through exocytosis [50]. Another study reported that SLPI partially resides in the nucleus, where it competes with NFκB for binding sites at pro-inflammatory gene promoters [51]. However, in this study, we did not observe any nuclear translocation of fluorescently labeled AMWAP into microglia, even after prolonged incubation periods.

Our results showed that AMWAP effectively inhibited the rapid LPS-mediated proteolytic degradation of IRAK-1 and IκBα, concomitantly preventing nuclear NFκB translocation. Further experiments demonstrated that IκBα phosphorylation and ubiquitination were not influenced by AMWAP. This is in agreement with previous studies reporting similar observations for SLPI and elafin in monocytes [31,32]. Our experiments also revealed that AMWAP did not exert direct inhibitory effects on 20S proteasome activity. In accordance with our data, the LPS-inducible antibacterial peptide PR39 was shown to block degradation of IκBα without affecting its phosphorylation, ubiquitination, or overall proteasome activity [52]. The anti-bacterial and anti-protease functions of whey acidic proteins are mainly attributed to their WAP domains [53-55]. In contrast, the suppression of the LPS-induced pro-inflammatory macrophage response mediated by SLPI is independent of its anti-protease activity [56]. Further analysis will be required to determine which functional elements in AMWAP are responsible for its immunomodulatory function.

AMWAP significantly inhibited the LPS-triggered release of nitric oxide from microglia, an effect that has been observed in SLPI overexpressing macrophages [39,56]. AMWAP also reduced microglial neurotoxicity on 661W photoreceptor cells. In line with this, SLPI has been shown to reduce LPS-induced neutrophil apoptosis and a lack of SLPI resulted in increased apoptosis of myeloid cells [57,58].

Filopodia formation is a hallmark of homeostatic microglial cells that constantly survey their microenvironment with their branched protrusions [5]. Our experiments indeed showed that AMWAP promotes the formation of branched filopodia. High phagocytic clearance of apoptotic debris by microglia is also regarded as an important anti-inflammatory feature [59,60]. We observed an increased phagocytic uptake of apoptotic photoreceptor fragments by AMWAP-treated microglia, which was independent of further LPS stimulation as apoptotic cells *per se* represent an activating stimulus for microglia [8]. Along this line, macrophages show elevated secretion of SLPI during the ingestion of cellular debris, and a similar effect can be hypothesized for AMWAP and microglia [61]. Of note, elafin overexpression protects macrophages from neutrophil elastase-mediated degradation of their receptors for apoptotic cell recognition, effectively restoring phagocytic recognition and clearance in inflammatory settings [62].

Reactive microglia generally exhibit higher proliferation rates compared to regulatory microglia [9,60]. However, we were not able to detect any anti-mitotic effect of AMWAP in BV-2 microglia. It is noteworthy, that there is conflicting evidence in the literature regarding the anti-proliferative potential of whey acidic proteins. One report even observed a SLPI-induced increase of stem cell proliferation towards an oligodendroglial cell fate via induction of cyclin D1 [63].

## Conclusions

We have shown that AMWAP is a secreted anti-inflammatory protein of reactive microglia that acts in a paracrine fashion. Endocytosed AMWAP is localized in a perinuclear region where it prevents NFκB signaling via inhibition of IRAK-1 and IκBα proteolytic degradation. AMWAP does not affect phosphorylation or ubiquitination of these signaling proteins and has no influence on overall 20S proteasome activity. On a functional level, AMWAP inhibits pro-inflammatory gene expression, reduces microglial neurotoxicity, promotes filopodia formation, and increases phagocytic uptake of apoptotic debris. In conclusion, our data suggest that AMWAP triggers a neuroprotective phenotype in microglia and that whey acidic proteins may be potential therapeutics to inhibit a detrimental microglial phenotype in neurodegenerative diseases of the brain and retina.

## Additional files

Additional file 1: Figure S1. AMWAP overexpression reduces LPS-induced nuclear translocation of NFκB p65. (A) BV-2 microglia overexpressing AMWAP-GFP and MOCK-GFP control cells were incubated with 50 ng/ml LPS for 2 h before anti-NFκB immunocytochemistry. AMWAP-GFP overexpressing cells showed a reduced nuclear translocation of NFκB p65 as shown by quantification of nuclear and cytosolic fluorescence signals (B) Data show mean ± SD (*n* = 9/group) **P* < 0.05, ***P* < 0.01, ****P* < 0.001. Scale bar = 20 μm. Additional file 2: Figure S2. AMWAP inhibits LPS-induced turnover of (phosphorylated) IκBα. Control and AMWAP-treated BV-2 microglia were incubated with 50 ng/ml LPS for 30 min, and cytosolic protein extracts were prepared before immunoblot analysis of phosphorylated IκBα. LPS-induced IκBα phosphorylation was observed in AMWAP-treated and control cells. However, AMWAP-treated cells accumulated phosphorylated IκBα, protecting it from proteasomal degradation and even inhibited the basal IκBα turnover in unstimulated cells. GAPDH served as loading control. pIκBα, phosphorylated inhibitor of kappa B alpha.
