# Supplementary figures and images for: Activated microglia/macrophage whey acidic protein (AMWAP) inhibits NFκB signaling and induces a neuroprotective phenotype in microglia
Source: J Neuroinflammation. 2015 Apr 19;12:77. doi: 10.1186/s12974-015-0296-6 (PMC4417279; doi:10.1186/s12974-015-0296-6)

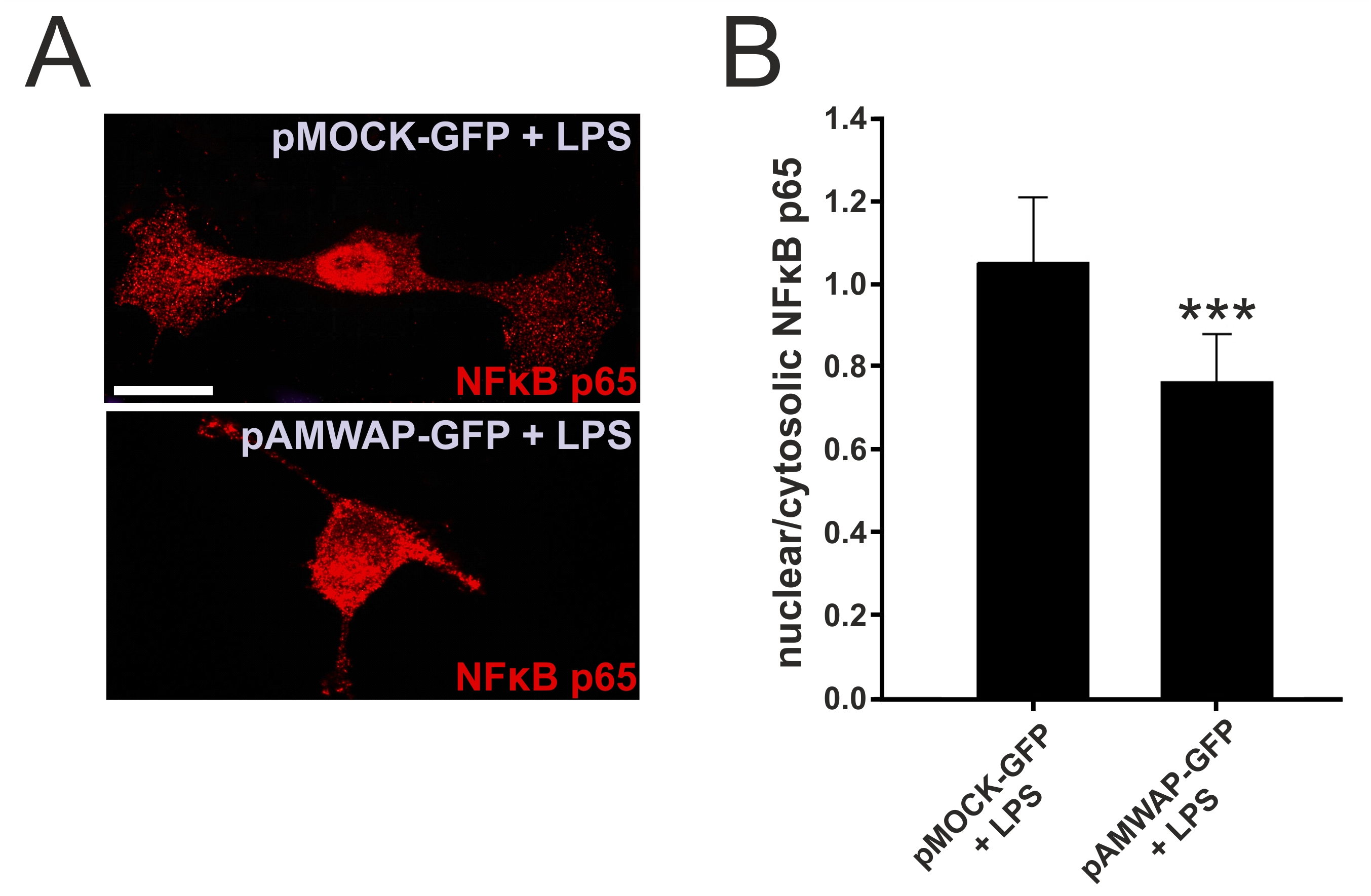

Supplement: Additional file 1: Figure S1. — AMWAP overexpression reduces LPS-induced nuclear translocation of NFκB p65. (A) BV-2 microglia overexpressing AMWAP-GFP and MOCK-GFP control cells were incubated with 50 ng/ml LPS for 2 h before anti-NFκB immunocytochemistry. AMWAP-GFP overexpressing cells showed a reduced nuclear translocation of NFκB p65 as shown by quantification of nuclear and cytosolic fluorescence signals (B) Data show mean ± SD (n = 9/group) *P < 0.05, **P < 0.01, ***P < 0.001. Scale bar = 20 μm. [file 12974_2015_296_MOESM1_ESM.jpg]

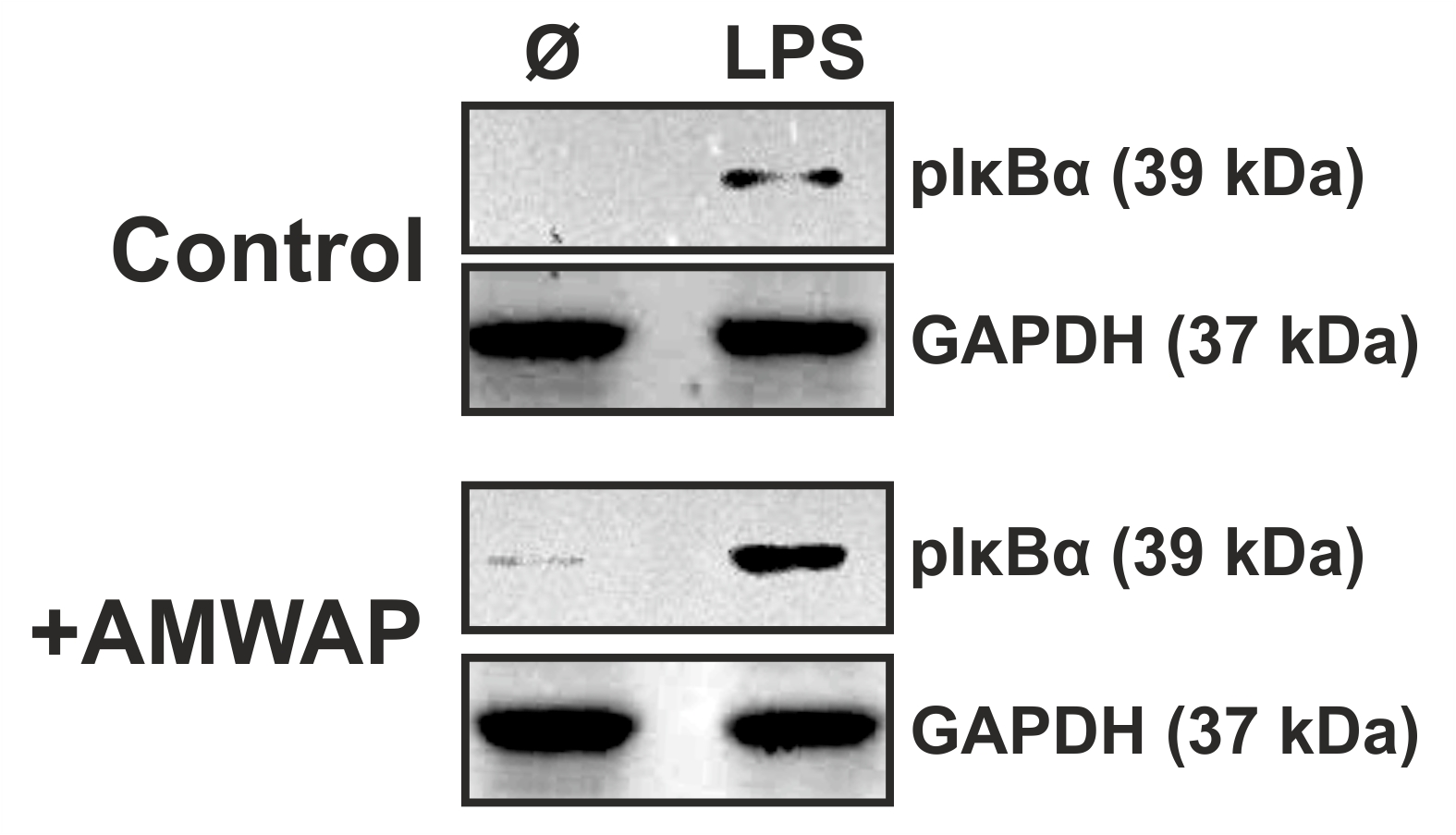

Supplement: Additional file 2: Figure S2. — AMWAP inhibits LPS-induced turnover of (phosphorylated) IκBα. Control and AMWAP-treated BV-2 microglia were incubated with 50 ng/ml LPS for 30 min, and cytosolic protein extracts were prepared before immunoblot analysis of phosphorylated IκBα. LPS-induced IκBα phosphorylation was observed in AMWAP-treated and control cells. However, AMWAP-treated cells accumulated phosphorylated IκBα, protecting it from proteasomal degradation and even inhibited the basal IκBα turnover in unstimulated cells. GAPDH served as loading control. pIκBα, phosphorylated inhibitor of kappa B alpha. [file 12974_2015_296_MOESM2_ESM.jpg]
